# The radiation of New Zealand’s skinks and geckos is associated with distinct viromes

**DOI:** 10.1186/s12862-024-02269-4

**Published:** 2024-06-13

**Authors:** Stephanie J. Waller, Richelle G. Butcher, Lauren Lim, Kate McInnes, Edward C. Holmes, Jemma L. Geoghegan

**Affiliations:** 1 Department of Microbiology and Immunology, University of Otago, Dunedin, 9016 New Zealand; 2 Tāwharau Ora, School of Veterinary Science, Massey University, University Avenue, Fitzherbert, Palmerston North, 4442 New Zealand; 3 Department of Conservation, P.O. Box 10420, Wellington, 6143 New Zealand; 4 School of Medical Sciences, The University of Sydney, Sydney, NSW 2006 Australia; 5 Institute of Environmental Science and Research, Wellington, New Zealand

**Keywords:** Virome, Metatranscriptomics, New Zealand, Skink, Gecko, Viral co-divergence, Host radiation

## Abstract

**Background:**

New Zealand is home to over 120 native endemic species of skinks and geckos that radiated over the last 20–40 million years, likely driven by the exploitation of diverse habitats formed during the Miocene. The recent radiation of animal hosts may facilitate cross-species virus transmission, likely reflecting their close genetic relationships and therefore relatively low barriers for viruses to emerge in new hosts. Conversely, as animal hosts adapt to new niches, even within specific geographic locations, so too could their viruses. Consequently, animals that have niche-specialised following radiations may be expected to harbour genetically distinct viruses. Through a metatranscriptomic analysis of eight of New Zealand’s native skink and gecko species, as well as the only introduced lizard species, the rainbow skink (*Lampropholis delicata*), we aimed to reveal the diversity of viruses in these hosts and determine whether and how the radiation of skinks and geckos in New Zealand has impacted virus diversity and evolution.

**Results:**

We identified a total of 15 novel reptilian viruses spanning 11 different viral families, across seven of the nine species sampled. Notably, we detected no viral host-switching among the native animals analysed, even between those sampled from the same geographic location. This is compatible with the idea that host speciation has likely resulted in isolated, niche-constrained viral populations that have prevented cross-species transmission. Using a protein structural similarity-based approach, we further identified a highly divergent bunya-like virus that potentially formed a new family within the *Bunyavirales*.

**Conclusions:**

This study has broadened our understanding of reptilian viruses within New Zealand and illustrates how niche adaptation may limit viral-host interactions.

**Supplementary Information:**

The online version contains supplementary material available at 10.1186/s12862-024-02269-4.

## Background

Viruses of squamates are understudied [1]. There are approximately 12,000 reptilian species on Earth [2] that likely harbour a vast array of viruses yet to be discovered. New Zealand is home to at least 77 endemic skink and 48 endemic gecko species [3, 4], along with a single species of introduced skink, the rainbow skink (*Lampropholis delicata*), believed to have been established in New Zealand from Australia during the 1960s [5, 6]. Several of New Zealand’s skink and gecko species are still awaiting formal classification and new taxa continue to be identified [3]. New Zealand’s lizard taxa are unique since all endemic species have evolved in probable isolation for millions of years. Consequently, New Zealand lizard species have evolved several unique characteristics. For example, 99% of New Zealand’s lizards exhibit viviparity (i.e. produce live offspring) [7], although only around 19% of lizard taxa found around the world are viviparous [8]. The high incidence of viviparity exhibited by New Zealand’s native lizards is believed to have helped these species adapt to living in cold climates since better incubation conditions in utero can promote successful embryonic development in comparison to oviparity where eggs are laid [7, 8].

Based on both DNA and fossil evidence, it is believed that skinks colonised New Zealand from New Caledonia during the early Miocene (16–22.6 million years ago (mya)) following the Oligocene drowning (∼25 mya) via long-distance overwater dispersal [9, 10]. Skinks began to rapidly speciate during the mid to early Miocene into clear open-habitat, forest and coastal radiations [9]. Consequently, all of New Zealand’s native skinks are currently considered to fall within a single genus, the *Oligosoma* alongside the Lord Howe Island skink (*Oligosoma lichenigerum*), which can be found on Philip Island and Lord Howe Island [9]. Similarly, New Zealand geckos form a single monophyletic group that diverged from the Australian sister group of geckos approximately 40.2 mya after the formation of the Tasman Sea [11]. Following this divergence event, New Zealand geckos experienced an extensive radiation, particularly during the mid to late Miocene, resulting in seven related genera: *Mokopirirakau*, *Toropuku*, *Dactylocnemis*, *Woodworthia*, *Tukutuku*, *Naultinus* and *Hoplodactylus* [11]. The rapid radiation experienced by both skinks and geckos is likely attributed to the fragmented landscape of New Zealand following the Oligocene drowning, resulting in the isolation and evolution of independent populations of lizards [12]. Additionally, during the Miocene, New Zealand experienced an increase in land area due to geographic changes resulting in an increase in habitat diversity that further promoted lizard radiation [9, 11, 12]. As a result of these geographic and climatic changes, only around 7% of New Zealand’s lizard species are considered habitat generalists, although high levels of lizard sympatry can be observed within certain New Zealand habitats [13].

Despite their ubiquity, very little is known about the viruses carried by skinks and geckos globally [14]. Paramyxoviruses have previously been detected in healthy skinks at London Zoo [15], and papillomaviruses have been identified in multiple species of healthy geckos located on Christmas and Cocos Islands [16]. Adenoviruses and iridoviruses have been associated with disease in both skinks and geckos [17, 18], while metatranscriptomic surveys of seemingly healthy skinks and geckos have uncovered a vast array of virus diversity, including viruses from the families *Arenaviridae*, *Astroviridae*, *Caliciviridae*, *Iridoviridae*, *Amnoonviridae*, *Bornaviridae*, *Picornaviridae*, *Rhabdoviridae*, *Adomaviridae*, *Hantaviridae* and *Flaviviridae* [19, 20].

Since both New Zealand’s skinks and geckos have experienced radiations, albeit across vastly different timeframes, they provide a way to explore how host macroevolution may have impacted virus evolution. Herein, we sampled cloacal swabs from eight native New Zealand skink and gecko species as well as the introduced rainbow skink, sampling nine species from four locations including both wild and captive populations. We used a metatranscriptomic approach to provide an initial survey of the viromes of these species to determine how host radiation may have impacted viral diversity and evolution. We also explored whether viruses in skinks and geckos were distinct due to their different evolutionary histories, introduction times and ecology, and whether viruses had jumped from introduced rainbow skinks to native lizards.

## Materials and methods

### Skink and gecko cloacal swab sample collection

Wild introduced rainbow skinks (*L. delicata*), pacific geckos (*Dactylocnemis pacificus*), copper skinks (*Oligosoma aeneum*), moko skinks (*O. moco*), ornate skinks (*O. ornatum*), Suter’s skinks (*O. suteri*), raukawa geckos (*Woodworthia maculata*, also known as common geckos) and Northern spotted skinks (*O. kokowai*) were caught using either a pitfall [21], cell foam retreat [22], artificial cover object [22], hand capture, or gecko house method [22] across three different locations in New Zealand (Shakespear Regional Park, Rangitoto Island and Matiu/Somes Island) in 2021. Matiu/Somes Island is an off-shore island that is free from introduced lizard species, while Shakespear Regional Park and Rangitoto Island are home to both native skinks and geckos and the introduced rainbow skink. In addition, a captive population of Kapitia skinks (*O. salmo*) that were rescued from the West Coast of the South Island of New Zealand in 2018 and relocated to Auckland Zoo before Cyclone Fehi destroyed a large proportion of their one-kilometre coastal habitat were also captured and sampled in 2021 [23], having spent four years in captivity. More information regarding sample locations, species, and the number of individual skinks and geckos caught at each sampling site is provided in Supplementary Table 1. While the number of species and locations sampled in this study were limited, this sampling approach allowed us to undertake an initial survey of the viruses present in nine of New Zealand’s skink and gecko species located across four sampling sites. Due to the nature of working with *taonga* (treasured by Māori, the indigenous population of New Zealand) and endangered species, sampling was necessarily minimally invasive. Cloacal swabs were placed into 1 mL of RNA stabilisation solution (RNAProtect Tissue Reagent, Qiagen) and were stored at 4 °C until the samples were sent to the University of Otago within one week of sampling, where they were stored at -80 °C until RNA was extracted.

### Total RNA extraction of skink and gecko cloacal swab samples

Frozen cloacal swabs were defrosted before being placed in ZR BashingBead Lysis Tubes (0.1 mm and 0.5 mm) (Zymo Research) filled with 1 mL of DNA/RNA shield (Zymo Research). Lysis tubes were placed into a mini-beadbeater 24 disruptor (Biospec Products Inc.) and were homogenised for five minutes. RNA was extracted using the ZymoBIOMICS MagBead RNA kit (Zymo Research) with a few additions to the manufacturers protocol. Briefly, three additional molecular grade pure ethanol washes were undertaken to remove any residual guanidine contamination. RNA was quantified using a nanodrop. RNA from 285 cloacal swabs were at suitable concentrations for sequencing. Equal volumes of RNA from 4–36 individuals were pooled into 16 groups based on species and location (see Supplementary Table 1).

### RNA sequencing

Extracted RNA was subject to total RNA sequencing. Libraries were prepared using the Illumina Stranded Total RNA Prep with Ribo-Zero Plus (Illumina) and 16 cycles of PCR. Paired-end 150 bp sequencing of the RNA libraries was performed on the Illumina NovaSeq 6000 platform using a single S4 lane.

### Virome assembly and virus identification

Paired reads were trimmed and assembled de novo using Trinity v2.11 with the “trimmomatic” flag option and default settings [24]. Sequence similarity searches against a local copy of the NCBI nucleotide (nt) database (2021) and the non-redundant protein database (2021) using nt Basic Local Alignment Search Tool (BLASTn) and Diamond (BLASTx), respectively, were used to annotate assembled contigs with a maximum expected value of 1 × 10^–5^ and more sensitive alignment mode selected [25]. Contigs were categorised into kingdoms using the BLASTn and Diamond (BLASTx) “sskingdoms” flag option. Non-viral BLAST hits including host contigs with sequence similarity to viral transcripts (e.g. endogenous viral elements) were removed from further analysis during manual screening. A maximum expected value of 1 × 10^–10^ was used as a cut-off to filter putative viral contigs. Viral contigs that have previously been identified as viral contaminants from laboratory components were also removed from further analysis [26]. Based on the BLASTn and Diamond results (database accessed June 2023), putative viral contigs were further analysed using Geneious Prime 2022.2.2 to find and translate open reading frames (ORFs).

Near complete viral sequences discovered using this approach were used in instances as a reference to run the raw reads from other libraries against it to obtain more complete polymerase sequences using Bowtie2 [27].

### Protein structure similarity search for viral discovery

Similar to previous work [28], we used a protein structure similarity search to identify highly divergent viral transcripts, termed orphan contigs, that did not share significant amino acid sequence similarity to other known transcripts. Such “orphan contigs” [28] were translated into ORFs using the EMBOSS getorf program [29], with the minimum nt size of the ORF set to 1,000 nt, the maximum nt size of the ORF set to 50,000 and the “methionine” flag option set to only report ORFs with a methionine amino acid starting codon. Reported ORFs were submitted to Phyre2, which uses remote homology detection to build 3D models to predict and analyse protein structure and function [30]. Virus sequences with predicted polymerase structures with a confidence value of > 90% were aligned with representative amino acid sequences from the same viral order obtained from NCBI RefSeq using MAFFT v7.490. Conserved domains were visually confirmed before phylogenetic trees were estimated using the same method outlined in the Mateirals and Methods Sect. Virus phylogenetic analysis.

### TSA mining for highly divergent novel bunya-like viruses

To identify other highly divergent novel bunya-like viruses we screened the protein sequence of the Raukawa gecko associated bunya-like virus (PP272801) against transcriptome assemblies available in NCBI’s Transcriptome Shotgun Assembly (TSA) sequence database [31] using the translated BLAST tool. Searches were restricted to eukaryotes (taxid:2759). Putative viral sequences were analysed using Geneious Prime 2022.2.2 to find and translate ORFs. Translated protein sequences were then queried against the online non-redundant protein database (database accessed November 2023) using the BLASTp tool.

### Estimating viral transcript abundance estimations

Viral abundances were estimated using Trinity’s “align and estimate” tool. RNA-seq by expectation–maximization [32] was selected as the method of abundance estimation, Bowtie2 [27] as the alignment method and the “prep reference” flag enabled. To mitigate the impact of contamination due to index-hopping, viral transcripts with expected abundances of less than 0.1% of the highest expected abundance for that virus across other libraries were removed from further analysis. Total viral abundance estimates for viruses from vertebrate hosts (i.e. skinks or geckos) across viral families and orders were compiled across all libraries. Estimated abundances per million reads were standardised to the number of paired reads per library.

### Virus phylogenetic analysis

Translated viral protein polymerase sequences (i.e., RNA-dependent RNA polymerase (RdRp), replicase, DNA polymerase or nonstructural protein 1) were aligned with representative protein sequences from the same virus family or order obtained from NCBI RefSeq as well as the closest BLASTp hit using MAFFT v7.490 [33]. Ambiguously aligned regions were removed using trimAL v1.2rev59 with the gap threshold flag set to 0.9 [34]. IQ-TREE v1.6.12 was used to estimate maximum likelihood phylogenetic trees for each viral species/family/order [35]. The LG amino acid substitution model was selected with 1000 ultra-fast bootstrapping replicates. Phylogenetic trees were annotated using Figtree v1.4.4 [36]. Only those viruses that were likely to directly infect skinks or geckos, based on their phylogenetic position on the tree (i.e., were related to other vertebrate viruses), were analysed. The only exception to this was the highly divergent bunya-like virus that was identified through protein structural analysis. All other invertebrate host and environmental associated viruses (i.e., that were likely components of host diet or microbiome), that were closely related and phylogenetically positioned beside previously described invertebrate or environmental viruses, were removed from further analysis.

### Bipartite network analysis and plots

All plots were created in R v4.3.1 using RStudio v2021.09.1 with the tidyverse ggplot2 package [37]. A bipartite network analysis was used to investigate the relationships of viral families that were or were not shared between skink and gecko species. The bipartitie network was created using the ggbipart package [38] within the ggplot2 environment [37]. A heatmap was created to compare the relative viral family abundance percentages across libraries. Viral family abundance estimates per million reads were first standardised by the number of raw reads in each library. Standardised viral family abundance estimates were then normalised across each library and a log-scale heatmap was created using the geom_tile function [37]. Dual plots were also generated to compare the average viral family richness across lizard species and the average viral abundance per 100 million reads across lizard species to the average raw reads across libraries of lizard species. The standard deviation of the data was also calculated and is represented by the error bars on the plots.

### Viral nomenclature

A virus was tentatively considered a novel species if it shared < 90% amino acid similarity within the most conserved region (i.e. RdRp/polymerase) [39, 40]. For novel virus sequences we have provided a provisional virus (common) name prior to formal verification by the International Committee on Taxonomy of Viruses (ICTV).

## Results

**Fig. 1:**
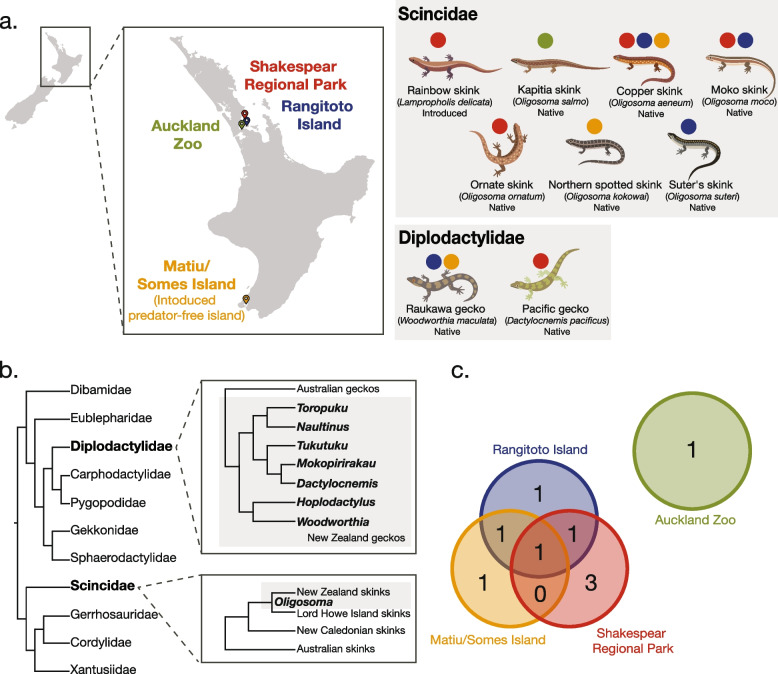
**a** Map of New Zealand indicating the four sites where skinks and geckos were sampled. **b** Cladogram illustrating the evolutionary relationship of geckos in the family Diplodactylidae compared to skinks in the family Scincidae [41], and the evolution of New Zealand skinks and geckos [9, 11]. **c** Venn diagram depicting the number of skink and gecko species shared between the four sampling sites

A total of nine species of skinks and geckos were sampled across four different sites in the North Island of New Zealand (Fig. 1). Wild copper skinks were the only species sampled across all three ‘wild population’ sites, while the captive Kapitia skinks were the only species sampled from Auckland Zoo (Fig. 1c). Total RNA from 285 individuals were pooled into 16 libraries based on species and sample location (see Fig. 1 and Supplementary Table 1). The number of sequencing reads that were generated from the skink and gecko metatranscriptomic libraries varied between 36 – 72 million paired-end reads per library (Supplementary Table 1). The percentage of viral reads ranged from 0.0005% to 1.17% across the 16 libraries (Supplementary Table 1).

### Viral abundance and diversity

**Fig. 2:**
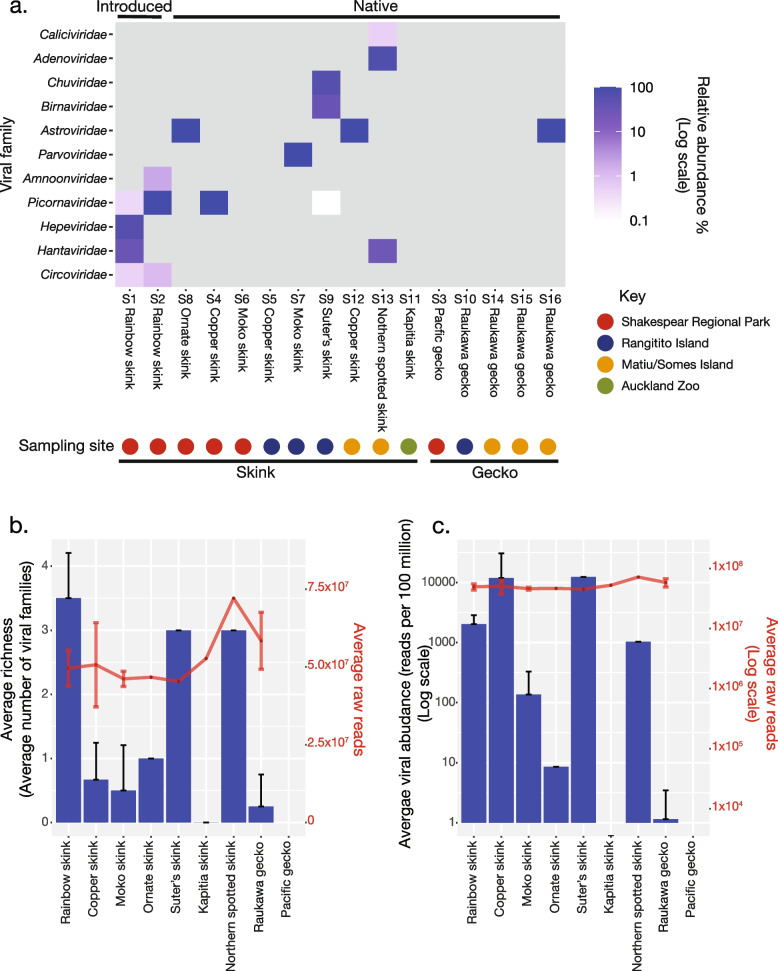
**a** Heatmap of relative viral abundance (%) across libraries. Information regarding pooled RNA sampling sites is denoted by coloured dots and in the key, while information regarding introduced vs native species and host species is noted above and below the heatmap respectively. **b** Dual plot of average viral family richness across lizard species (left y-axis) and average raw reads across libraries of lizard species (right y-axis). Error bars represent standard deviation values. **c** Log scale dual plot of average viral abundance (reads per 100 million) across lizard species (left y-axis) and average raw reads across libraries of lizard species (right y-axis). Error bars represent standard deviation values

Analysis of skink and gecko metatranscriptomes revealed viral transcripts spanning 11 different viral families that likely infect lizard hosts based on their sequence similarity (Fig. 2a). Seven out of the 16 skink and gecko libraries contained no viruses that appeared to be directly infecting skinks and geckos, including captive Kapitia skinks from Auckland Zoo (Fig. 2a and b). Consequently, it was not feasible to statistically compare the differences in virome composition between captive and wild populations of skinks and geckos, or the differences in virome composition between skinks and geckos generally.

Rainbow skinks, the only introduced species, had the highest viral richness, with virus transcripts spanning five viral families (Fig. 2a) and had an average viral family richness of 3.5 (Fig. 2b). Only two of these families (*Hantaviridae* and *Picornaviridae*) were also found in native skinks and geckos while the others (*Circoviridae*, *Hepeviridae* and *Amnoonviridae*) were unique to rainbow skinks (Fig. 2a). Overall, skinks appeared to have more diverse viromes than geckos as transcripts from 11 viral families were identified in skinks compared to transcripts from only one viral family identified in geckos (Fig. 2a and b). Similarly, the average viral abundance appeared to be higher in skinks than geckos, ranging from 0–12,324 reads per 100 million for skinks to 0–1.1 reads per 100 million for geckos (Fig. 2c). However, caution needs to be taken given that there were only four sequencing libraries of geckos sampled compared to 11 libraries of skinks, such that there may be random variation within the data. Notably, skink and gecko species sampled from the same locations had different viromes (Fig. 2a).

### Viral network

**Fig. 3:**
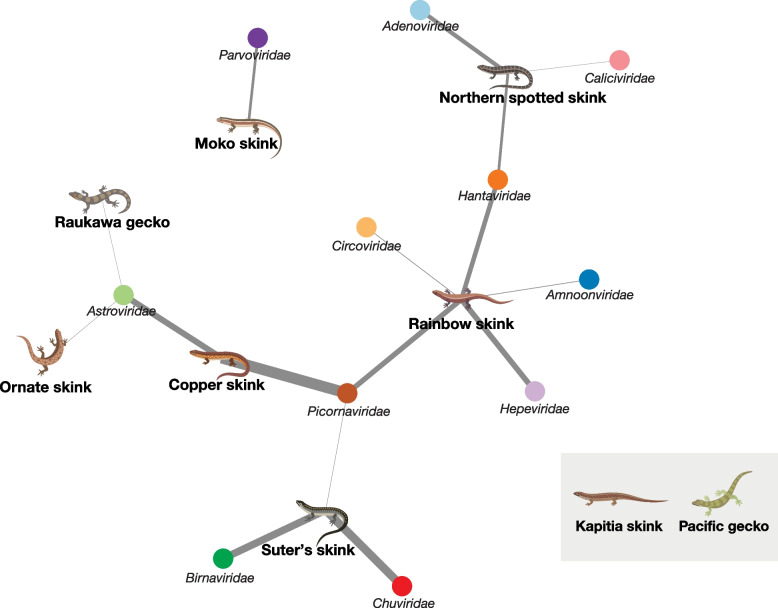
Bipartite network depicting the connections between viral families shared between skinks and geckos sampled. Branch thickness is weighted by the standardised abundance in reads per million of viral transcripts within a given host species

A bipartite network analysis revealed that picornaviruses and astroviruses were sampled more frequently among hosts sampled than any other viral family detected (Fig. 3). Captive Kapitia skinks and wild Pacific geckos did not harbour any viruses besides those likely associated with their diet or environment. Moko skinks were disconnected from the rest of the bipartite network since only parvovirus transcripts were identified in this species, and this virus was absent from other sampled hosts. In addition, transcripts from the *Birnaviridae*, *Chuviridae*, *Circoviridae*, *Hepeviridae*, *Amnoonviridae*, *Caliciviridae* and *Adenoviridae* viral families were not shared between more than one host, indicating unique virome composition among hosts.

### Negative-sense RNA viruses in skinks

#### Amnoonviridae

**Fig. 4:**
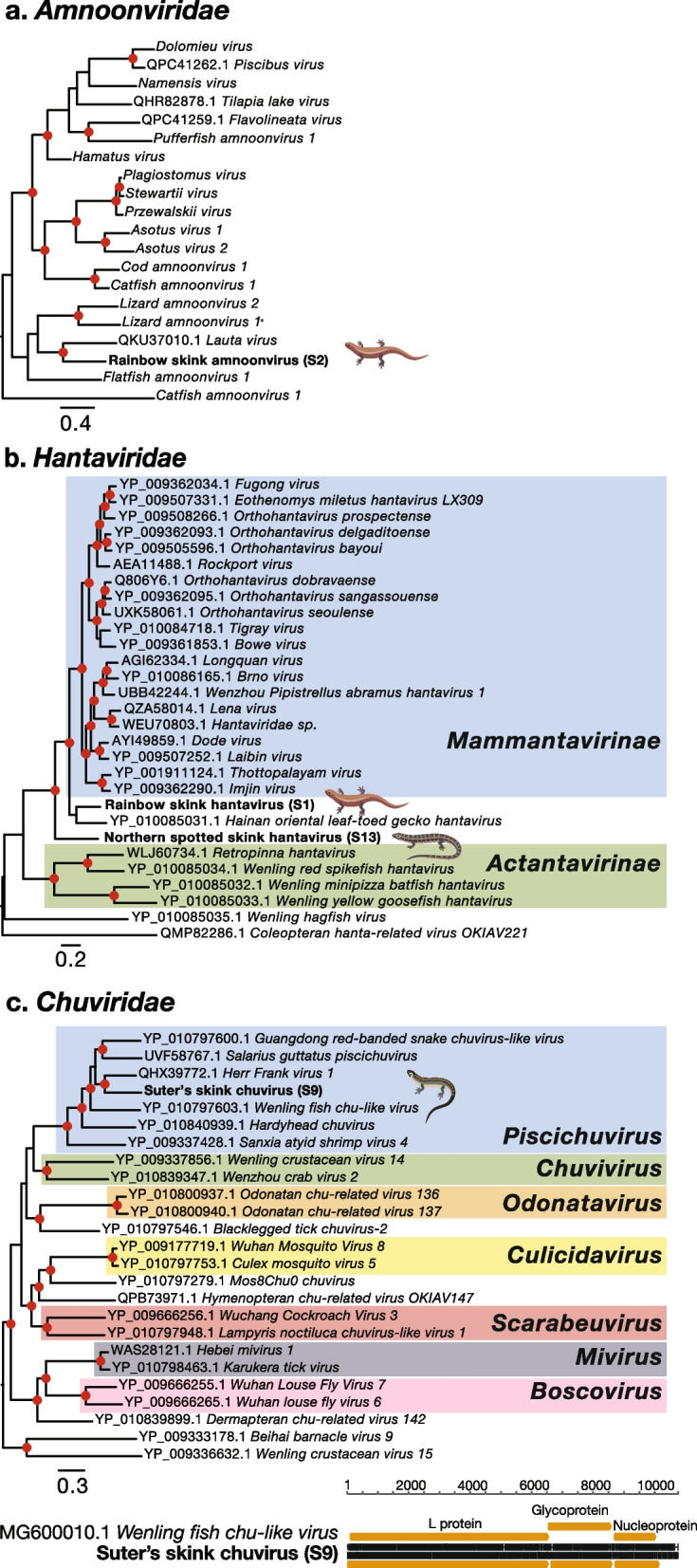
Phylogenetic trees of negative-sense RNA viruses. Maximum likelihood phylogenetic trees of representative viral transcripts containing the RdRp from negative-sense RNA viral families (**a**) *Amnoonviridae*, (**b**) *Hantaviridae* and (**c**) *Chuviridae*. Skink viruses identified in this study are bolded while known genera and subfamilies are highlighted. Branches are scaled to the number of amino acid substitutions per site. All phylogenetic trees were midpoint-rooted. Nodes with ultrafast bootstrap values of > 70% are noted by a red circle. If near full-length genomes of skink viruses were uncovered a nt alignment (black) and the predicted ORFs (orange) of the skink virus along with a representative complete viral genome from the same family is shown below the respective phylogenetic trees

A single amnoonvirus was identified in rainbow skinks from Shakespear Regional Park, provisionally named rainbow skink amnoonvirus (Fig. 4a). Rainbow skink amnoonvirus shared 39% amino acid sequence similarity with the closest known genetic relative, *Lauta virus* (QKU37010.1) which was previously identified in the liver of a healthy Australian gulf tree gehyra (*Gehyra lauta*), a species of gecko [28].

#### Hantaviridae

Hantaviruses were identified in northern spotted skinks from Matiu/Somes Island and rainbow skinks from Shakespear Regional Park. Northern spotted skink hantavirus shared 44% amino acid sequence similarity to *Orthohantavirus seoulense* (UXK58061.1) which was previously identified in the lung of a brown rat (*Rattus norvegicus*) from South Korea (Fig. 4b) [42]. Comparatively, Rainbow skink hantavirus shared 68% amino acid sequence similarity with *Fugong virus* (YP_009362034.1) identified in kachin red-backed voles (*Eothenomys eleusis*) from China [43]. Phylogenetically, both novel skink viruses fell as a sister group to the *Mammantavirinae*, clustering instead with *Hainan oriental leaf-toed gecko hantavirus* (YP_010085031.1) sharing 40–64% amino acid sequence similarity. *Hainan oriental leaf-toed gecko hantavirus* was previously identified in oriental leaf-toed geckos (*Hemidactylus bowringii*) from China (Fig. 4b).

#### Chuviridae

A virus from the *Chuviridae* was identified in Suter’s skinks from Rangitoto Island. Suter’s skink chuvirus shared 53% amino acid sequence similarity with *Herr Frank virus 1* (QHX39772.1) identified in boa constrictors (*Boa constrictor constrictor*) from Brazil that had boid inclusion body disease associated with reptarenaviruses (*Arenaviridae*) [44]. Suter’s skink chuvirus along with *Herr Frank virus 1* fell within the *Piscichuvirus* genus (Fig. 4c). A full-length 10,633 nt genome of Suter’s skink chuvirus was uncovered, encoding three ORFs; L protein, glycoprotein and nucleoprotein (Fig. 4c).

### Positive-sense RNA viruses in skinks and geckos

#### Caliciviridae

**Fig. 5:**
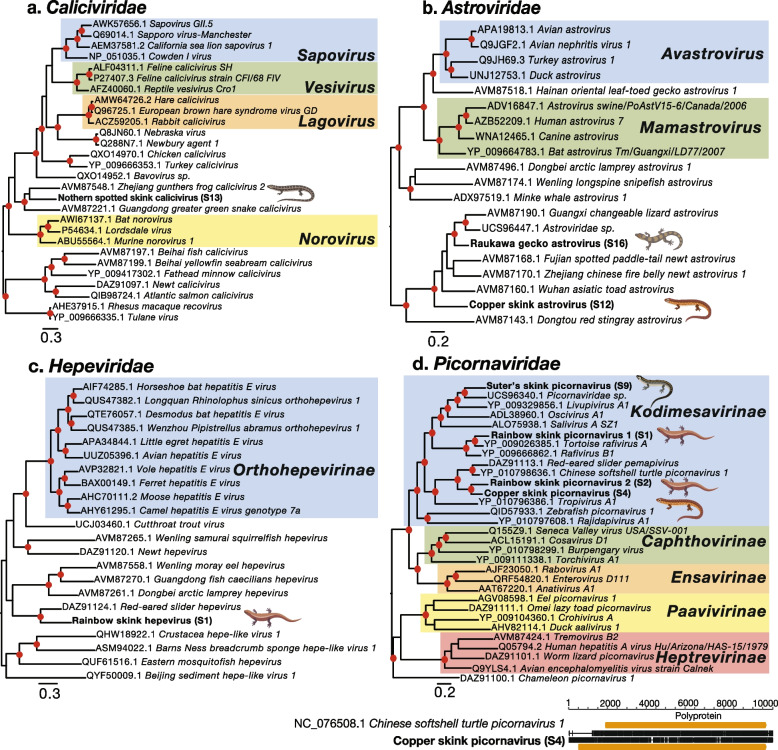
Phylogenetic trees of positive-sense RNA viruses. Maximum likelihood phylogenetic trees of representative viral transcripts containing the RdRp from positive-sense RNA families (**a**) *Caliciviridae*, (**b**) *Astroviridae*, (**c**) *Hepeviridae*, (**d**) *Picornaviridae*. Skink and gecko viruses identified in this study are bolded while known genera and subfamilies are highlighted. Branches are scaled to the number of amino acid substitutions per site. All phylogenetic trees were midpoint rooted. Nodes with ultrafast bootstrap values of > 70% are noted by a red circle. If near full-length genomes of skink or gecko viruses were uncovered a nt alignment (black) and the predicted ORFs (orange) of the skink or gecko virus along with a representative complete viral genome from the same family is shown below the respective phylogenetic trees

We identified Northern spotted skink calicivirus from animals sampled on Matiu/Somes Island. This virus was most closely related to *Zhejiang gunthers frog calicivirus 2*, although sharing only 38% amino acid sequence similarity. *Zhejiang gunthers frog calicivirus 2* was previously identified in Günther’s frogs (*Sylvirana guentheri*) from China (Fig. 5a) [45].

#### Astroviridae

An astrovirus was identified in raukawa geckos from Matiu/Somes Island (Fig. 5b). Raukawa gecko astrovirus shared 68% amino acid sequence similarity with *Zhejiang Chinese fire belly newt astrovirus 1* (AVM87170.1), which was identified in a Chinese fire belly newt (*Cynops orientalis*) from China [45]. Phylogenetically, this virus also fell closely beside *Astroviridae sp.* (UCS96447.1) previously uncovered in the faeces of Theobald's toad-headed agamas (*Phrynocephalus theobaldi*) from China [46] and *Guangxi changeable lizard astrovirus* (AVM87190.1) previously identified in the gut of oriental garden lizards (*Calotes versicolor*) from China (Fig. 5b) [45].

A second astrovirus was identified in copper skinks from Matiu/Somes Island (Fig. 5b). Copper skink astrovirus shared 52% amino acid sequence similarity also with *Astroviridae sp.* (UCS96447.1) [46], but following phylogenetic analysis this virus was most closely related to *Wuhan asiatic toad astrovirus* (AVM87160.1) identified in asiatic toads (*Bufo gargarizans*) from China (Fig. 5b) [45]. A third astrovirus was also identified in the metatranscriptomic library of ornate skinks from Shakespear Regional Park, although only a fragment of the capsid protein was recovered. Consequently, we were unable to conduct a phylogenetic analysis of this virus.

#### Hepeviridae

A virus from the *Hepeviridae* was identified in rainbow skinks from Shakespear Regional Park (Fig. 5c). This virus was termed Rainbow skink hepevirus and shared 52% amino acid sequence similarity with *Red-eared slider hepevirus* (DAZ91124.1), which was identified in red-eared slider turtles (*Trachemys scripta elegans*) from China [20].

#### Picornaviridae

Four picornaviruses from the *Kodimesavirinae* subfamily were identified across three skink species (Fig. 5d). Rainbow skink picornavirus 1 from Shakespear Regional Park shared 76% amino acid sequence similarity to *Tortoise rafivirus A* (YP_009026385.1) identified in Forsten's tortoises (*Indotestudo forsteni*) from USA experiencing severe disease including anorexia, diarrhea and mucosal ulcerations associated with *Sulawesi tortoise adenovirus 1* [47]. An additional picornavirus, Rainbow skink picornavirus 2, was found in the same host species and same location but was genetically similar to *Red-eared slider pemapivirus* (DAZ91113.1) [20], sharing 56% amino acid sequence similarity. Copper skink picornavirus, from copper skinks sampled from the same location, also shared 56% amino acid sequence similarity to *Red-eared slider pemapivirus* and was 68.37% similar to Rainbow skink picornavirus 2. The high sequence similarity shared between the Copper skink picornavirus and the Rainbow skink picornavirus 2 likely indicates cross-species transmission in the evolutionary past, particularly as these species were both sampled from Shakespear Regional Park. A full-length 9,830 nt genome of Copper skink picornavirus was uncovered (Fig. 5d). A fourth picornavirus, Suter’s skink picornavirus, was also identified from Suter’s skinks from Rangitoto Island (Fig. 5d). Suter’s skink picornavirus shared 52% amino acid sequence similarity with *Picornaviridae sp.* (UCS96340.1) previously identified in the faeces of Theobald's toad-headed agama from China [46].

### DNA viruses in skinks and geckos

#### Circoviridae

**Fig. 6:**
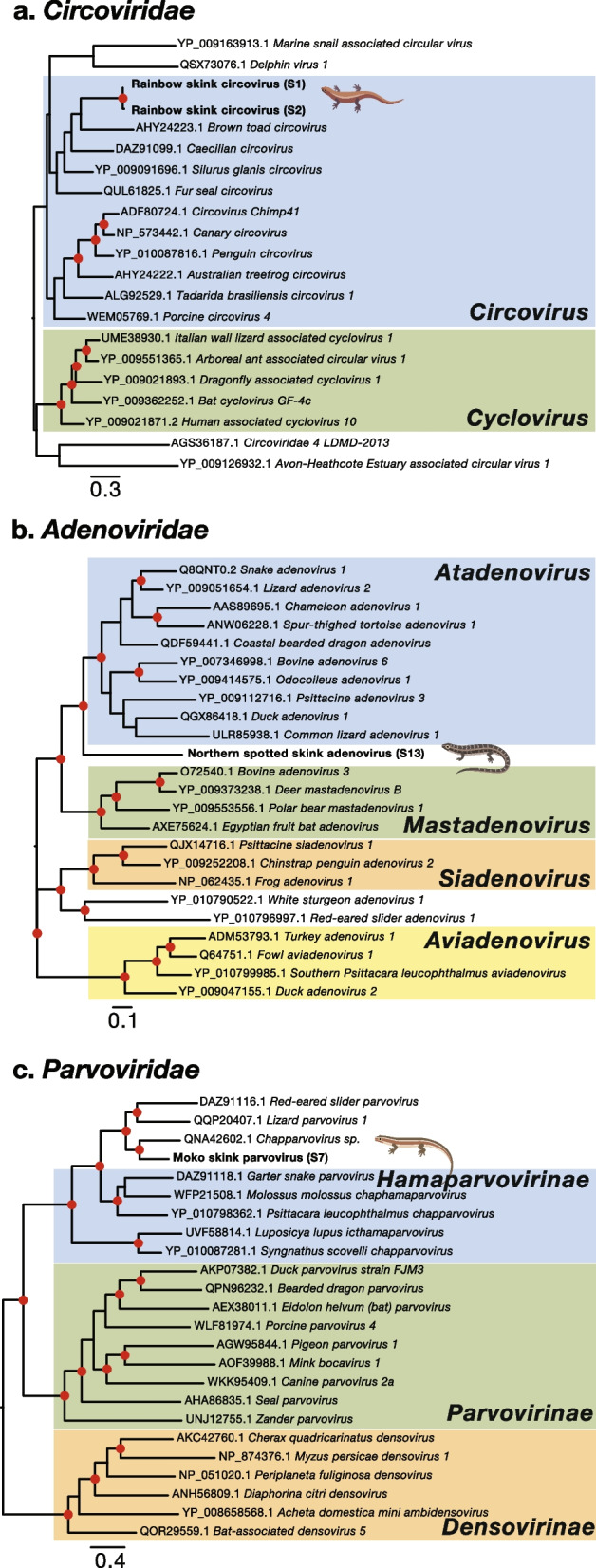
Phylogenetic trees of DNA viruses. Maximum likelihood phylogenetic trees of representative viral transcripts containing the replicase, DNA polymerase or nonstructural protein 1 from the families (**a**) *Circoviridae*, (**b**) *Adenoviridae* and (**c**) *Parvoviridae*. Skink and gecko viruses identified in this study are bold while known genera and subfamilies are highlighted. Branches are scaled to the number of amino acid substitutions per site. All phylogenetic trees were midpoint rooted. Nodes with ultrafast bootstrap values of > 70% are noted by a red circle

Two libraries of rainbow skinks from Shakespear Regional Park had replicase transcripts from the *Circoviridae* sharing > 98% amino acid sequence similarity (Fig. 6a). Rainbow skink circovirus shared 61% amino acid sequence similarity with *Delphin virus 1* (QSX73076.1) identified in orcas (*Orcinus orca*) from Saint Vincent and the Grenadines [48] but phylogenetically this virus fell more closely beside *Brown toad circovirus* (AHY24223.1) sampled from common toads (*Bufo bufo*) from Hungary (Fig. 6a) [49].

#### Adenoviridae

An adenovirus was identified in northern spotted skinks from Matiu/Somes Island (Fig. 6b). Northern spotted skink adenovirus shared 51% amino acid sequence similarity to *Duck adenovirus 1* (QGX86418.1) identified in dead ducks from Canada [50], although this virus fell as a sister group to members of the genus *Atadenovirus*.

#### Parvoviridae

Moko skinks from Rangitoto Island carried Moko skink parvovirus (Fig. 6c). This virus shared 52% amino acid sequence similarity with *Chapparvovirus sp.* (QNA42602.1) identified in both diseased and healthy central bearded dragons (*Pogona vitticeps*) from Australia [51].

### Novel viruses identified through protein structural similarity

A protein structure similarity based search was used to identify highly divergent novel viruses from contigs that did not share clear sequence similarity to other known sequences (i.e., “orphan contigs”). We identified a total of 5,947,316 orphan contigs across skink and gecko metatranscriptomic libraries contributing to 57.86% and 45.75% of the total contigs identified within the 11 skink and five gecko cloacal metatranscriptomic libraries, respectively. Of these, 9,254 contained a translatable ORF of 1000–50,000 nt in length. These were submitted to Phyre2 [30] for structural similarity analysis, resulting in the detection of one putative viral RdRp structure with a confidence of > 90%. A 2,793 nt orphan contig from raukawa geckos from Matiu/Somes Island had an identifiable structural relative: an RdRp structure from *Severe Fever with Thrombocytopenia Syndrome Virus* (Protein Data Bank identifier 8asb), a member of the *Pheuniviridae* (order *Bunyavirales*), with a confidence of 93% and 22% percentage identity.

**Fig. 7:**
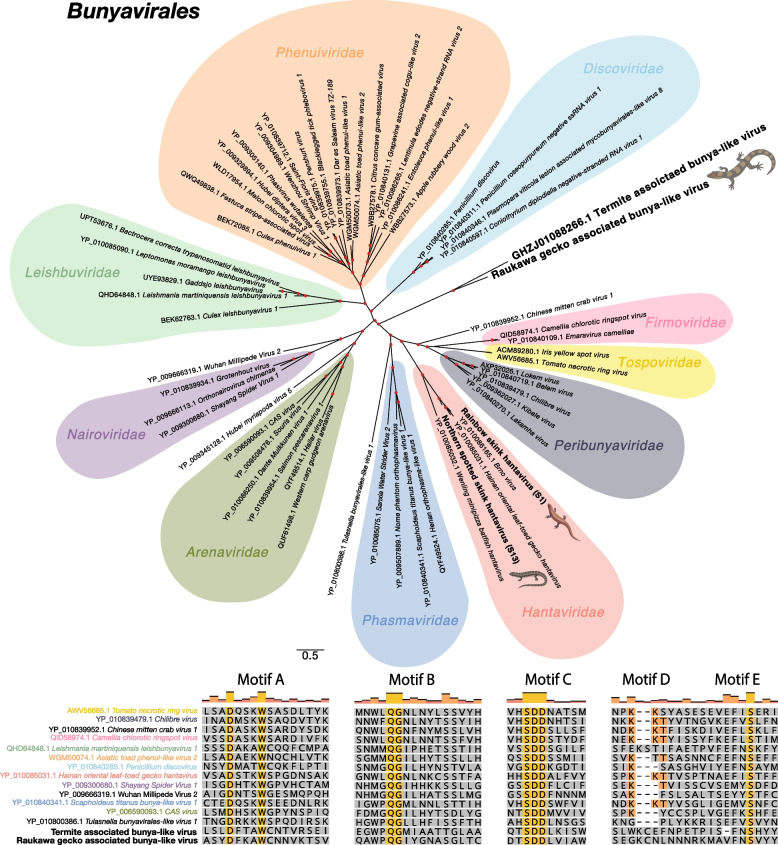
Maximum likelihood unrooted phylogenetic tree of representative viral transcripts containing the RdRp from the *Bunyavirales*. Viruses identified in this study and the termite virus identified by screening the TSA are bolded while families are highlighted. Branches are scaled to the number of amino acid substitutions per site. Nodes with ultrafast bootstrap values of > 70% are noted by a red circle. Below the phylogeny is an alignment of *Bunyavirales* RdRp amino acid sequences. Conserved *Bunyavirales* A-E RdRp motifs are shown while the Raukawa gecko associated bunya-like virus and the Termite associated bunya-like virus are bolded

We next screened this highly divergent protein sequence against the TSA database and identified a 6,597 nt-long transcript from *Coptotermes acinaciformis* (GHZJ01088266.1), a species of subterranean termites native to Australia sharing only 27% amino acid sequence similarity with the novel virus found in raukawa geckos. This contig had a 2,119 amino acid long ORF, although there were no significant matches when this protein sequence was queried against the non-redundant protein database using BLASTp. Order-level phylogenetic analysis of both viruses revealed a potentially novel and divergent viral family within the *Bunyavirales* (Fig. 7a). Although highly divergent at the amino acid sequence level, both Raukawa gecko associated bunya-like virus and Termite associated bunya-like virus contained the highly conserved *Bunyavirales* A, B, C and E sequence motifs (Fig. 7b) [52]. Given the phylogenetic clustering, it is likely that Raukawa gecko associated bunya-like virus is associated with dietary and environmental hosts.

## Discussion

We characterised the cloacal viromes of nine different species of skinks and geckos sampled across four different sites located on the North Island of New Zealand. In total, we identified 15 novel skink and gecko viruses spanning 11 viral families as well as one highly divergent bunya-like virus, which is unlikely to be directly infecting its reptilian host but potentially represents a new viral family within the *Bunyavirales*. All viruses identified in this study were novel (i.e., shared < 90% amino acid similarity to their relatives), further highlighting the uncharacterised viral diversity present in reptiles [20], particularly in New Zealand.

Of note, we found no evidence of viral host-jumping among native skinks and geckos even though both groups have diversified within New Zealand. The only potential host-jumping event detected was within the *Picornaviridae* between the only introduced species – the rainbow skink – and native copper skinks. The relatively high sequence similarity (~ 70%) between rainbow skink and copper skink picornaviruses suggests a viral host-jumping event has occurred since rainbow skinks colonised New Zealand, particularly as both species were sampled from the same location. Drivers of skink and gecko radiation include changes in climate, habitat and geography [9, 11], resulting in over 120 species identified to date with further species yet to be characterised [3]. While preliminary, our results suggest that the diverse open habitat, forest and coastal niches occupied during host speciation may have resulted in largely unique viromes among skinks and geckos sampled here [9, 53]. Indeed, it is striking that even different species sampled from the same sites had markedly different virome compositions. Expanding sampling to encompass a broader array of New Zealand’s lizard species, geographical locations, including sites in the South Island, and across time, would enhance our ability to make comprehensive conclusions and allow us to identify whether the limited host-jumping observed here is also observed nationally across all New Zealand lizard populations.

Our results are in contrast with those previously seen in African cichlid fishes that experienced a rapid adaptive radiation over the past 10 million years [54], albeit the present study was much more limited in size. Viral host-jumping among African cichlid species was frequent across multiple different viral families, likely due to their genetic similarity and therefore host cell receptors, decreasing the barriers to viral host-switching [54]. While habitat separation of cichlid fish was effective at driving host speciation, viral transmission was evidently not prohibited.

The seemingly infrequent cross-species transmission between introduced rainbow skinks and native skinks and geckos can be considered a positive finding from a conservational standpoint. The introduction of exotic species can have a devastating impact on native populations due to several factors such as predation, competition or the introduction of pathogens [55]. Native species from island ecosystems such as New Zealand are particularly at risk of disease as many have been isolated for millions of years and are often immunologically naïve to potential pathogens [56]. Introduced rainbow skinks harboured the highest viral richness (five viral families), greater than any other skink or gecko species. This high viral richness may be attributed to their gregarious nature and their tendency to lay eggs in large communal nests [5, 57], increasing opportunities for viral transmission although further sampling would be required to reinforce such patterns.

Nearly half of the skink and gecko cloacal swab metatranscriptome libraries (seven of the 16), contained no *bona fide* lizard viruses, such that we were unable to statistically compare skink and gecko virome composition. Similarly, no viruses were identified in a previous study that sampled Chalky Island skinks (*O. tekakahu*) located on an off-shore island in New Zealand [58]. However, it is important to note that our ability to identify vertebrate host-associated viruses may have been limited due to the use of cloacal swabs, particularly for viruses that exhibit tissue specific tropisms [59]. However, it might also be the case that very few viruses were present in these hosts when they colonised New Zealand [60].

It is also possible that some lizard viruses, or more likely those associated with the diet and/or microbiome of lizards, are so divergent that they may not be detected using sequence similarity approaches [61], particularly as New Zealand’s endemic flora and fauna have been isolated for millions of years [62]. Nevertheless, one highly divergent virus was identified using protein structural homology. Raukawa gecko associated bunya-like virus shared protein structural similarities to *Severe Fever with Thrombocytopenia Syndrome Virus* (within the *Phenuiviridae*). Subsequent exploration of published transcriptomes led to the identification of a viral transcript within *Coptotermes acinaciformis*, a species of subterranean termite native to Australia, that shared sequence similarity to this divergent virus. Phylogenetic analysis revealed that both viruses did not cluster within any of the currently classified families within the *Bunyavirales*, indicating that they likely represent a new viral family. Host assignment of these viruses remains unclear although they are likely invertebrate in origin. These results further highlight the benefits of sampling undersampled species such as reptiles.

## Conclusions

This study serves as an introductory exploration into the viromes of New Zealand’s lizards that have historically been under-sampled. We documented the viromes of nine of New Zealand’s skink and gecko species. In doing so, we identified 15 novel skink and gecko viruses, in turn enhancing our understanding of the viruses that circulate within New Zealand’s lizard populations. The limited cross-species viral transmission between skink and gecko species sampled here may reflect the rapid radiation of New Zealand skinks and geckos during the Miocene that resulted in niche-constrained viral populations. Further sampling of a wider range of New Zealand lizard taxa and geographic locations over multiple time points are required to better understand these viral dynamics.

### Supplementary Information

Supplementary Material 1: Supplementary Table 1. Data surrounding skink and gecko library pooling and sampling site locations.

Supplementary Material 2: Supplementary Table 2. Data surrounding the viral contigs that were identified in this study and the top BLASTp results.

## Data Availability

The raw sequencing reads generated in this project are available on the Aotearoa Genomic Data Repository, DOI number 10.57748/H447-H585 while viral sequences have been submitted to GenBank under the accession numbers PP272786—PP272802 (Supplementary Table S2). Alignments and code for the virome analysis can be found at https://github.com/stephwaller/NZ-Skink-Gecko-Virome-Paper.git.

## Abbreviations

BLAST: Basic Local Alignment Search Tool
ICTV: International Committee on Taxonomy of Viruses
mya: Million years ago
nt: Nucleotide
ORF: Open Reading Frame
RdRp: RNA-dependent RNA polymerase
TSA: Transcriptome Shotgun Assembly
